# Changes in Training, Lifestyle, Psychological and Demographic Factors, and Associations With Running-Related Injuries During COVID-19

**DOI:** 10.3389/fspor.2021.637516

**Published:** 2021-06-07

**Authors:** Hillary H. Holmes, Patrick G. Monaghan, Kamden K. Strunk, Max R. Paquette, Jaimie A. Roper

**Affiliations:** ^1^School of Kinesiology, Auburn University, Auburn, AL, United States; ^2^Department of Educational Foundations, Leadership and Technology, Auburn University, Auburn, AL, United States; ^3^College of Health Sciences, University of Memphis, Memphis, TN, United States

**Keywords:** COVID-19, injury, environment, cross-country (XC), track and field, surface, intensity

## Abstract

The primary purpose of this study was to examine how the type and magnitude of changes in running behavior, as a consequence of COVID-19 pandemic restrictions, influence running-related injuries. Secondarily, we aimed to examine how lifestyle and psychosocial well-being measures may influence running behavior change. An online survey was advertised to individuals over the age of 18 that currently run or have previously participated in running for exercise. The survey questions examined injury history and new injuries sustained during COVID-19 restrictions, as well as changes related to training behavior changes, training environment changes, social behaviors, and psychosocial well-being. Changes reflected differences in running behaviors prior to COVID-19 restrictions (1 month prior to COVID-19 restrictions being imposed) and during COVID-19 restrictions (May 5 to June 10, 2020). A total of 1,035 runners were included in the analysis. Current injuries sustained during COVID-19 occurred in 9.5% of the runners. Injured runners made a greater number of total changes (*p* = 0.031) as well as training-related (*p* = 0.042) and environment-related (*p* = 0.017) changes compared with uninjured runners. A significant relationship was found between injury and those that reported less time to exercise to changes in work environment (*p* = 0017). This study highlights the multi-dimensional nature of running-related injuries and the need to consider the interaction of multiple changes in running behavior, rather than isolating single factors. Greater understanding of the underlying causes of running-related injuries can help reduce the risk of future injury.

## Introduction

Running is one of the most accessible and popular forms of exercise, requiring minimal equipment and facilities. Over 60 million people worldwide participated in running in 2017 (Ham et al., 2009; Bureau of Labor Statistics, U.S. Department of Labor, The Economics Daily, 2016). Importantly, physical activity and running can protect and enhance physical, mental, and social components of health (Greist et al., 1979; Chan and Grossman, 1988; Penedo and Dahn, 2005; Eime et al., 2013; Xie et al., 2020). Running has been related to a 30 to 45% lower risk of mortality and a 45 to 70% lower risk of cardiovascular disease-related mortality (Lee et al., 2017). Not only can running benefit physical health but also psychological health is reported to be higher in runners and worsens when habitual runners are prevented from running (Chan and Grossman, 1988; Trost et al., 2002). Maintaining the accessibility of running is essential for achieving optimal physical and mental health benefits.

One threat to maintaining optimal running for exercise is sustaining a running-related injury (RRI), a common risk at all levels of running. In novice and recreational runners, RRI rates per 1,000 h of running range from 7 to 79.3% (Blair et al., 1987; Jakobsen et al., 1994; van Gent et al., 2007; Buist et al., 2010; Videbæk et al., 2015; Hespanhol Junior et al., 2016; Bertelsen et al., 2017). This broad range of RRI incidence may highlight inadequacies in identifying RRI risk factors. Some research indicated that specific running behaviors, like high training distance, frequencies, and intensities, are associated with running RRIs (Nielsen et al., 2012; Ramskov et al., 2018, Hulme et al., 2017). However, results remain inconsistent, with some studies that indicated no association with higher training distance and RRI, and others that reported higher training distance to be protective against RRIs (Wen et al., 1998; Kelsey et al., 2007). Changes in running behaviors also have had conflicting evidence in how they associate with RRIs (Johnston et al., 2003; Nielsen et al., 2014,?; Hulme et al., 2017). One study indicated that 25% of individuals that started a running routine sustained an RRI within their first 37 km of running (Nielsen et al., 2014). Training progressions limited to 10% weekly increases in running duration or intensity have commonly and anecdotally been proposed to minimize injury, but with inconclusive evidence (Johnston et al., 2003; Nielsen et al., 2014). One study indicated differences in injury risk between 10 and 30% with increases in weekly running distance (Nielsen et al., 2014), while another research has reported that injured runners, on average, increased weekly running distances by 32%, whereas uninjured runners increased by 22% (Nielsen et al., 2013). Inconsistencies in the validity of this recommendation could result from the same increase in external load factors, like running distance, and, thus, lead to different increases in internal mechanical load factors (e.g., tissue strain and stress) and physiological response (e.g., perceived effort), especially in the presence of fatigue (Johnston et al., 2003; Nielsen et al., 2014; Paquette et al., 2020). Therefore, it is likely that running injuries are not exclusively influenced by a change in a single training-related factor such as distance.

Indeed, to comprehensively understand RRIs, it could be less pertinent to assess isolated running behaviors and more important to identify the influence of multiple changes in a more complex framework (Bertelsen et al., 2017). For example, one study identified that 53% of injured runners made variations in running behaviors (velocity, distance, volume, and frequency) compared with 32% of uninjured runners (Ferreira et al., 2012). This complex framework can extend past running-related behaviors and into factors like physical, social, and psychological environments that influence human behavior. Thus, to better understand how multiple changes in running behavior influence RRI, it is also crucial to consider how psychological, social, and environmental factors may influence running behaviors (Paluska and Schwenk, 2000; Trost et al., 2002). For example, running offers positive social interaction, to help individuals form and maintain social relationships and promote social identity (Shipway et al., 2012; Robinson et al., 2014; Hindley, 2020; Xie et al., 2020). Engagement in physical activity, such as running, has also long been shown to be an effective treatment for social isolation, social anxiety, and depression (Petruzzello et al., 1993; Paluska and Schwenk, 2000; Barber et al., 2005; Blumenthal et al., 2012; Anderson and Shivakumar, 2013; Liu et al., 2015). Furthermore, environmental factors like access to facilities and safe running environments have impacted activity behavior, with the Centers for Disease Control and Prevention previously reporting a significant positive association between perceived neighborhood safety and physical activity (From the Centers for Disease Control and Prevention, 1999). For example, activity levels in urban areas have been significantly higher than in rural areas (Bauman et al., 1999; Brown et al., 1999; Brownson et al., 2000). It is plausible that fluctuations in an individual's psychological and social well-being, as well as environmental changes, may present an influence in running behaviors. Thus, knowing that alterations in running behaviors have potential to influence RRI risk, environmental, social, and psychological factors should be considered.

With the recent worldwide pandemic of coronavirus disease 2019 (COVID-19), individuals have been forced to alter their daily lifestyle and exercise behaviors due to changes such as closures of fitness facilities, changes in work and home obligations, and novel isolation protocols. For example, recent work studying youth long-distance runners found that COVID-19 social distancing restrictions impacted training habits (Bazett-Jones et al., 2020). Furthermore, recent studies have illustrated that physical activity levels during the COVID-19 global pandemic have decreased (Castañeda-Babarro et al., 2020; Gallè et al., 2020; Eek et al., 2021; Fearnbach et al., 2021; Puccinelli et al., 2021; Wilke et al., 2021). Self-reported moderate physical activity levels have been reported to decrease by 41%, while Eek and colleagues depicted that more than 65% of the participants reported changes in their physical activity (Eek et al., 2021; Wilke et al., 2021). Such forced changes from a known, habitual lifestyle to novel pandemic restrictions provided a unique scenario to investigate how new environmental, social, and psychological barriers influence running behaviors and RRI risk in a large sample. The primary purpose of this study was to identify (1) if COVID-19 restrictions influenced the magnitude, direction of change (increase, decrease, and no change), and total quantity of changes in running behavior (volume, intensity, surface type, route location, and running schedule) relate to RRIs, and (2) how lifestyle, psychological and demographic factors may influence changes in running behaviors due to COVID-19 restrictions. We first hypothesized that (1) larger magnitudes of change and more total changes in volume, intensity, route variability, and surface type would relate to RRIs, and (2) increased loneliness and negative affect may relate to RRIs.

## Materials and Methods

### Participants and Recruitment

Participants qualified for this study if they were over 18 years of age and if they were currently running for exercise. Participants also qualified if they were not currently running, but had recent, previous history of running within the past year. We aimed to collect these individuals with recent, previous history of running for exercise to identify if individuals stopped running due to COVID-19. This study was approved by Auburn University's Institutional Review Board (protocol number: 20-221 EX 2004), and only responses from participants who consented to participate were recorded and used for analysis. Participants were recruited through snowball sampling via social media platforms and running communities on social media platforms (Twitter, Instagram, Facebook), email, and word of mouth.

### Survey Development

An anonymous online survey was created and hosted on the survey platform Qualtrics (Qualtrics Labs Inc). Qualtrics is a university-approved and secure survey platform that allows participants to enter the survey through a link on their home browsers or mobile devices. The survey was created by three researchers experienced in data collection, running and locomotion research, and running exercise, allowing for comprehensive collaboration, distribution, and interpretation of the survey. All researchers and other researchers in respective laboratories looked over the survey for clarity. The survey was reviewed by three experts and pilot tested in 20 individuals similar to the target group to ensure clarity of questions and rating scales. No major changes were suggested.

### Survey Details

The survey included questions examining injury details, training-related changes, environment-related changes, social-related changes, and psychosocial well-being (Supplementary Material). To identify the changes in injuries and running behaviors, participants were asked in reference to before and after major COVID-19 lockdown restrictions in their geographical area. This survey was administered during the first COVID-19 lockdown procedures; meaning, individuals were largely transitioning from prior normalcy to first shift in lifestyle changes. Data reflecting “during COVID-19” were collected from May 5, 2020, through June 10, 2020, and data reflecting “before COVID-19” were collected 1 month prior to the introduction of COVID-19 restrictions. To explore changes in running behaviors that may have been imposed by environmental and social restrictions, questions reflected current behaviors (during COVID-19 restrictions) and behaviors in the month immediately before COVID-19 restrictions. Current behaviors were reflective of the period between May 5, 2020, and June 10, 2020, which included the presence of COVID-19 restrictions.

### Participant Demographics

The survey also contained questions that examined participant demographics, which can be found in Table 1. These included measures such as age, sex assigned at birth, race and ethnicity, and details concerning occupation status. Sex assigned at birth was included as per APA recommendations. Racial and ethnic categories were chosen to reflect and match census categories. Occupation status included examining the proportion of participants that reported as being in full-time or part-time employment, participants that reported as unemployed or seeking employment, and the proportion of participants that reported as students or retired. We also examined to what extent (completely, partially, or not changed) work had transitioned to a remote workplace.

**Table 1:** Participant demographics.

		**Uninjured**		**Injured**		**Total**	
		***N***	**(%)**	***N***	**(%)**	***N***	**(%)**
*N*		936	(90.40)	99	(9.60)	1,035	(100.00)
Age, M (SD)		35.86	(11.68)	36.10	(11.98)	35.88	(11.71)
Sex assigned at birth							
	Female	666	(71.20)	77	(77.80)	743	(71.80)
	Male	261	(27.90)	22	(22.20)	283	(27.30)
	Intersex	4	(0.40)	0	(0.00)	4	(0.90)
	Prefer not to say	2	(0.20)	0	(0.00)	2	(0.20)
Race							
	White	888	(94.90)	92	(92.90)	980	(94.70)
	Black or African American	10	(1.10)	1	(1.00)	11	(1.10)
	Asian	9	(1.00)	1	(1.00)	10	(1.00)
	American Indian or Native America/Native Hawaiian or Pacific Islander	29	(3.10)	5	(5.10)	34	(3.30)
Ethnicity							
	Hispanic or Latino	24	(2.60)	7	(7.10)	31	(3.00)
	Non-Hispanic or Latino	905	(97.40)	92	(92.90)	997	(96.30)
Occupation							
	Working full time	529	(56.50)	50	(50.50)	579	(55.90)
	Working part time	57	(6.10)	4	(4.00)	61	(5.90)
	Working self-employed	38	(4.10)	3	(3.00)	41	(4.00)
	Not working (temporary layoff)	59	(6.30)	6	(6.10)	65	(6.30)
	Not working (job seeking)	9	(1.00)	1	(1.00)	10	(1.00)
	Student	84	(9.00)	11	(11.10)	95	(9.20)
	Retired	33	(3.50)	3	(3.00)	36	(3.50)
	Other	127	(13.60)	21	(21.20)	148	(14.30)
Transition to remote workplace							
	Completely remote	432	(46.20)	41	(41.40)	473	(45.70)
	Partially remote	97	(10.40)	13	(13.10)	110	(10.60)
	No change	137	(14.60)	13	(13.10)	150	(14.50)
	Non-response	270	(28.80)	32	(32.30)	302	(29.20)

*Injured refers to individuals who sustained a new running-related injury (RRI) during COVID-19 restrictions. Uninjured refers to individuals who did not sustain a new RRI during COVID-19 restrictions*.

### Injury Details

All RRIs were self-reported using a list of common RRIs and the same definition of RRI: “Musculoskeletal pain in the lower limbs that causes a restriction on or stoppage of running (distance, speed, duration, or training) for at least 7 days or three consecutive scheduled training sessions, or that requires the runner to consult a physician or other health professional” (Messier et al., 2018). Participants were asked, “During COVID-19, have you gotten injured running?” If the participant selected “Yes,” they could select which injury from the same list of RRIs. If the participant selected “Yes” they were classified in the injured group, indicating that they had sustained a new RRI during COVID-19. If the participant selected “No,” they were classified in the uninjured group, indicating that they had not sustained a new RRI during COVID-19. There were options for participants to write in an injury that was not listed. Participants were also asked if they had a history of RRIs. The full list of injuries and questions can be found in the Survey Document in the Supplementary Material. Participants' history of RRIs and RRIs sustained during the study period were obtained.

### Training-Related Running Behavior

Questions investigating training-related running behaviors included examining the number of runs per week, weekly distance run, duration of the longest run per week, and the number of light, moderate, hard, or maximal intensity runs completed per week. The proportion of runs completed at each intensity was also calculated by dividing the number of runs at a particular intensity per week by the overall number of runs that week to provide an insight into the relative intensity load. Participants were asked to provide training-related running behaviors for the month leading up to the COVID-19 lockdown procedures in their geographic area and their training-related running behaviors currently during the first COVID-19 lockdown. The difference between current training-related running behaviors and before COVID-19 training-related behaviors was calculated to determine a change score, categorized as a decrease, increase, or no change for that specific running behavior.

### Environment-Related Running Behavior

Environment-related running behaviors included questions examining both running routes and running surface types. Participants were asked if they ran on mostly different routes, sometimes ran on different routes, or never ran on different routes before and during COVID-19 procedures. Participants were also asked how many times per week, they ran on different surface types before and during COVID-19 procedures. These surface types included the treadmill, inside and outside running tracks, outside rural and urban roads, outside on trails, and outside on the grass. Typical running routes and running surface types were asked regarding both the month leading up to COVID-19 restrictions and current environment-related running behaviors. Change scores, reflecting the difference between current and before COVID-19 environment-running behaviors were calculated to determine increases, decreases, or no change in particular running behaviors.

### Social-Related Running Behaviors

Social-related running behaviors before and during COVID-19 procedures included examining if participants ran on their own or with other people or objects. Participants were asked if they ran with members of their household, ran with teammates or a social group, ran alone inside on a treadmill, or ran alone. Responses were categorized into solidarity (ran alone inside on a treadmill and ran alone) and group-based running behaviors (they ran with members of their household, ran with teammates or a social group, ran with dog, and ran with stroller). Social-related running behaviors were asked both before COVID-19 restrictions and current social running behaviors during COVID-19. A shift from any group-based running behaviors to solidarity running behaviors was classified as a decrease in social-related running behaviors, and a shift from solidary running behaviors to group-based running behaviors was classified as an increase in social-related running behaviors.

### Psychosocial Well-Being Measures

The 20-item self-report Positive and Negative Affect Schedule (PANAS) (Watson et al., 1988) was used to measure psychological well-being. The PANAS measures emotions or feelings that an individual may experience and how they may influence actions and decision making. Positive affect refers to positive emotions and positive interactions with the environment, whereas negative affect refers to negative expressions and is associated with fear and sadness. The psychometric questionnaire contains 10 items that measure positive affect and 10 items that measure negative affect. Average positive and negative affect scores are derived from a five-point Likert scale that is used for scoring. The Likert scale offered responses of agreement, including “Very slightly or not at all, A Little, Moderately, Quite a bit, and Extremely” for each feeling or emotion, and participants were asked to “Indicate the extent you have felt this way during COVID-19.” The structure validity of the PANAS through confirmatory factor analyses have indicated overall good fit with minimal misspecification of the model regarding negative affect measures (Crocker, 1997). However, PANAS scores have previously been reported as having good internal consistency (positive affect α = 0.90, negative affect α = 0.91) and test–retest reliability (DeVellis, 2003; Serafini et al., 2016). The Three-Item Loneliness Scale, adapted from the Revised—UCLA Loneliness Scale (Russell et al., 1980) was used to measure the degree of loneliness and social isolation. Participants responded to three items indicating how often they felt a lack of companionship, felt left out, or felt isolated from others. Scores were summed for each item to garner an overall loneliness score, with higher scores indicating greater loneliness. The Three-Item Loneliness Scale has been shown to display good reliability (α = 0.72) and concurrent and discriminative validity, and depict strong correlations to the full and standard measure of loneliness, the R-UCLA (DeVellis, 2003; Hughes et al., 2004).

### Barriers

The survey also contained questions to examine what barriers running participants may or may not have experienced. Barriers may have been reflective of restrictions put in place to mitigate the spread of COVID-19, such as reduced access to workout facilities or arisen from changes in school and work environments resulting in increased obligations and decreased time for exercise. The survey also contained questions relating to potential psychological barriers such as apprehension in exercising and lack of motivation to exercise.

### Statistical Analyses

All survey responses are located in the Supplementary Table. Distance, frequency, and duration per week were reported in ranges (i.e., 0–10, 10–20 miles). Intensity and surface type were reported in number of days per week. Any increase or decrease in training, and environmental and social running behaviors, regardless of magnitude, was calculated from before to after COVID-19 isolation procedure (i.e., if a participant ran 0–10 miles before COVID-19 procedures and 10–20 miles during COVID-19 procedures, this was coded as an increase). Calculating proportions allowed interval data for statistical analysis. Data on intensity and surface types were collected in days out of the week (i.e., participant ran at a moderate intensity 2 days a week, or a participant ran on an outdoor track surface 2 days a week). Because we had discrete data and not data ranges for these outcome measures, we calculated proportions of intensity and surface type by the number of reported times per week running a certain intensity or on a certain surface divided by the total number of reported days (i.e., 2 days running at moderate intensity of a total 4 days running would equate to a proportion of 0.5). A change in proportions was calculated by the difference between the value during COVID-19 procedures and the value reported during the month prior to COVID-19 procedures. All statistical analyses were performed using IBM SPSS Statistics software program version 26 (SPSS Inc. Chicago, IL, USA), and level of significance was set to *p* < 0.05.

Runners were grouped into an injured group (indicating that they had sustained a new RRI since COVID-19 restrictions), and an uninjured group (indicating they had not sustained a new RRI since COVID-19 restrictions). For the purposes of comparing continuous scores (such as proportions, PANAS, and loneliness scores) between runners who had sustained and who had not sustained a new RRI during COVID-19, the Mann–Whitney U was conducted. This test is a non-parametric counterpart to the independent samples *t*-test and is more appropriate in cases of unbalanced design and where parametric assumptions are unlikely to be met (Strunk and Mwavita, 2020). In the present study, the group sizes were heavily imbalanced, making this test the more appropriate choice. For analyses testing association of categorical variables, such as yes/no questions, with injury status, we used chi-square tests of independence. This tests the independence of group membership on two categorical variables and is thus appropriate to those questions. An ordinary least squared (OLS) regression analysis was conducted to assess the relationship between PANAS and loneliness scores and changes in the various domains (training, environmental, and social) of running behavior. Finally, to test the association of continuous variables with one another, we used Pearson product-moment correlation coefficients.

## Results

### Participants

Data collection took place from May 5, 2020, through June 10, 2020. A total of 1,158 participants completed the survey; however, only 1,035 participants were included in the main analysis identifying associations among running behaviors, psychological well-being, and RRIs. One hundred nineteen participants were excluded as they reported that they were not currently running for exercise, and a further four participants were removed as their responses to open-ended items were clearly derogatory, non-sensical, or indicated they did not intend to provide a valid response. Of the participants who were not currently running for exercise, 13% (*n* = 16) had stopped running due to changes induced by COVID-19.

### Injury

During COVID-19, 9.5% of the participants sustained a new injury. Prior history of injury was associated with reported new RRIs (χ2_1_ = 20.396, *p* < 0.001), with those with a history of prior injuries overrepresented in the injured group [standardized residual (*SR*) = 3.10]. Of injured participants, 70.7% had history of prior injuries compared with only 46.7% of uninjured participants. Six percent of injured participants sustained a stress fracture or reaction type injury. Injuries were sustained to the foot (10.1%), ankle (10.1%), lower leg (24.2%) lower back (4%), pelvis or hip (10.1%), thigh (13.1%), and knee (13.1%). The types of injuries reported can be observed in Table 2.

**Table 2:** Reported running-related injuries sustained during COVID-19 restrictions.

**Reported injury**	**% Of Injured participants**
Achilles tendinopathy	9.1
Calf strains	17.2
Plantar fasciitis	4.0
Foot stress fracture	2.0
Foot unknown	4.0
Ankle sprain	7.1
Other ankle injury	3.0
Tibial stress fracture/reaction	3.0
Shin splints	2.0
Tibialis posterior tendinopathy	2.0
Patellar tendinopathy or pain syndrome	11.1
Knee meniscus injury	2.0
Iliotibial band syndrome	5.1
Hamstring strain	7.1
Other thigh muscular injury	4.0
Thigh femoral stress fracture/reaction	1.0
Groin strain	1.0
Hip unknown	2.0
Piriformis syndrome	4.0
Pelvis gluteus injury	2.0
SI joint dysfunction	1.0
Lower back	4.0

### Pre-COVID Running Behaviors

There were no significant differences in Pre-COVID training-related running behavior such as frequency (Z = −0.723, *p* = 0.470), distance (Z = −0.810, *p* = 0.418), duration (Z = −0.711, *p* = 0.477), light intensity (Z = −0.611, *p* = 0.541), moderate intensity (Z = −0.533, *p* = 0.594), hard intensity (Z = −1.023, *p* = 0.306), and maximal intensity (Z = −0.966, *p* = 0.334) between those that got injured and those that did not get injured. There was also no difference in the proportion of days training at light duration (Z = −1.255, *p* = 0.210), moderate (Z = −0.964, *p* = 0.335), hard (Z = −0.993, *p* = 0.321), or maximal (Z = −1.003, *p* = 0.316) intensities between the injured and uninjured. Furthermore, there was no significant difference in the proportion of days running on a particular surface type, including treadmill (Z = −0.363, *p* = 0.717), indoor track (Z = −0.715, *p* = 0.474), outdoor track (Z = −1.377, *p* = 0.168), rural road (Z = −0.415, *p* = 0.678), urban road (Z = −1.230, *p* = 0.219), trails (Z = −0.464, *p* = 0.643), or grass (Z = −0.637, *p* = 0.524) between the injured and uninjured runners.

### Changes in Categories of Running Behavior

**Figure 1:**
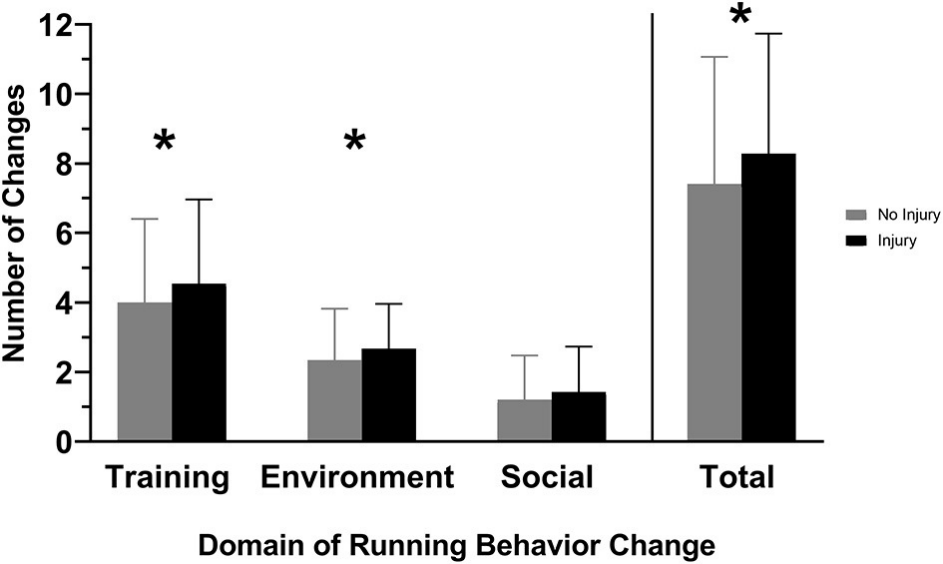
Injured refers to runners who sustained a new running-related injury (RRI) during COVID-19 restrictions. Uninjured refers to runners who did not sustain a new RRI during COVID-19 restrictions. Injured runners exhibited a greater number of changes in training and environment-related running behaviors, as well as cumulative overall changes. *p < 0.05.

We found that runners who sustained a new injury during COVID-19 made a significantly greater number of total running behavior changes (encompassing training, environment, and social factors) compared with uninjured runners (Z = −2.153, *p* = 0.031) (Table 3, Figure 1). Injured runners also exhibited significantly greater number of training (Z = −2.036, *p* = 0.042) and environmental (Z = −2.393, *p* = 0.017) changes in running behavior compared with uninjured runners. No significant difference between injured and uninjured runners were observed in the number of social changes in running behavior (Z = −1.712, *p* = 0.087).

**Table 3:** Means (and standard deviations) of total changes in running behaviors by injury group.

**Variable**	**Uninjured**	**Injured**	***p*-value**
Training changes	3.994 (2.411)	4.541 (2.421)	**0.042**
Environmental changes	2.345 (1.475)	2.674 (1.286)	**0.017**
Social changes	1.202 (1.273)	1.429 (1.301)	0.087
Total changes	7.408 (3.659)	8.281 (3.454)	**0.031**

*Injured refers to individuals who sustained a new RRI during COVID-19 restrictions. Uninjured refers to individuals who did not sustain a new RRI during COVID-19 restrictions*.

*Values in bold indicate significance (p < 0.05)*.

### Training-Related Running Behaviors

**Figure 2:**
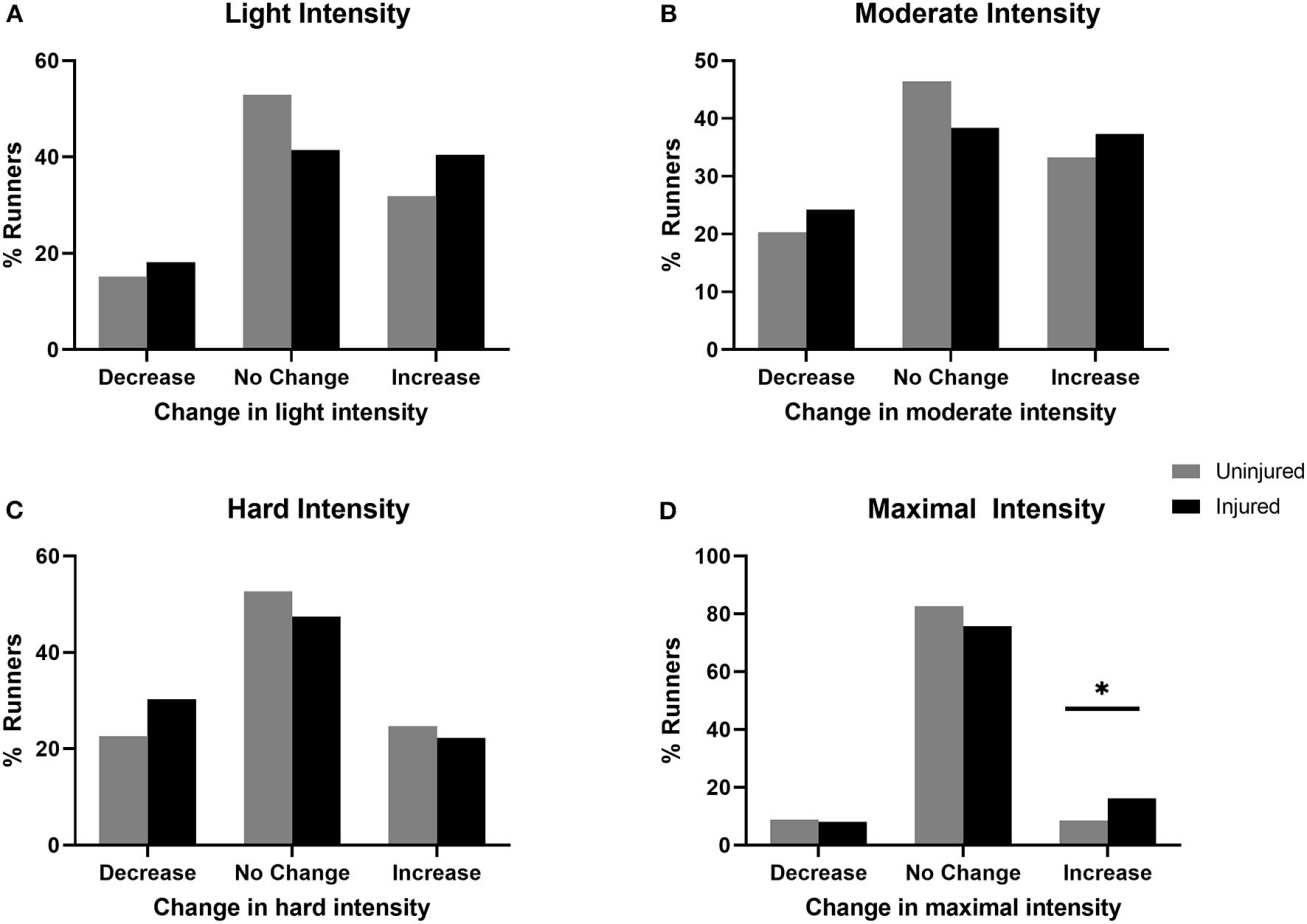
Injured refers to runners who sustained a new RRI during COVID-19 restrictions. Uninjured refers to runners who did not sustain a new RRI during COVID-19 restrictions. Changes in the number of days per week training at **(A)** light intensity, **(B)** moderate intensity, **(C)** hard intensity, and **(D)** maximal intensity. Chi-square test of independence revealed significant association between maximal intensity and injury group, with those increasing maximal intensity overrepresented in the injured runners. *p < 0.05.

**Figure 3:**
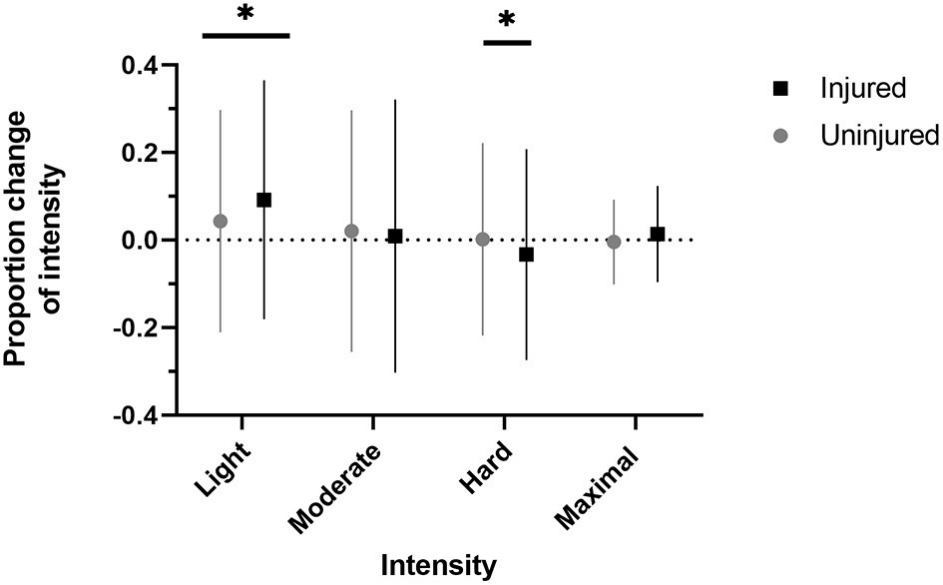
Injured refers to runners who sustained a new RRI during COVID-19 restrictions. Uninjured refers to runners who did not sustain a new RRI during COVID-19 restrictions. Change in the number of days spent running at a specific intensity proportional to total running intensities per week. Injured runners exhibited a relative increase in the proportion of light intensity runs and a relative decrease in the proportion of hard intensity runs. Dotted line indicates no change. *p < 0.05.

There was a significant relationship with changes in maximal intensity (χ2_2_ = 6.382, *p* = 0.041) with those increasing maximal intensity overrepresented in the group that experienced new injuries (SR = 2.289) (Figure 2). However, there was no significant relationship between those with and without new injuries with changes in distance run per week (χ2_2_ = 0.394, *p* = 0.821), days run per week (χ2_2_ = 0.507, *p* = 0.776), minutes of longest run (χ2_2_ = 1.505, *p* = 0.471), in light intensity (χ2_2_ = 4.807, *p* = 0.090), moderate intensity (χ2_2_ = 2.380, *p* = 0.304), hard intensity (χ2_2_ = 2.997, *p* = 0.223). When examining proportional changes in training-related running behaviors, there was a significant difference between injured and uninjured runners in proportional change in light intensity (Z = −2.296, *p* = 0.022) and hard intensity (Z = −2.565, *p* = 0.010) (Table 4, Figure 3). There was no significant difference between those with and without new injuries in proportional change in moderate intensity (Z = −0.063, *p* = 0.949) or maximal intensity (Z = −1.215, *p* = 0.224).

**Table 4:** Proportional changes in running intensity.

**Proportional change in intensity**	**Uninjured**	**Injured**	***p*-value**
Light intensity	**0.043 (0.254)**	**0.092 (0.273)**	**0.022**
Moderate intensity	0.020 (0.276)	0.009 (0.312)	0.949
Hard intensity	**0.001 (0.220)**	**−0.033 (0.241)**	**0.010**
Maximal intensity	–0.005 (0.097)	0.013 (0.110)	0.224

*Injured refers to individuals who sustained a new RRI during COVID-19 restrictions. Uninjured refers to individuals who did not sustain a new RRI during COVID-19 restrictions*.

*Values in bold indicate significance (p < 0.05)*.

*Means (and standard deviations) of proportional change in running intensity by injury group*.

### Environment-Related Running Behaviors

There was a significant relationship with outdoor trail running (χ2_2_ = 6.350, *p* = 0.012), where those with new injuries were overrepresented among those increasing outdoor trail running (SR = 1.9) and underrepresented among those with no change in outdoor trail running (SR = −1.4) (Table 5). There were no significant relationships with changes in indoor treadmill running (χ2_2_ = 0.151, *p* = 0.698), indoor track running (χ2_2_ = 0.850, *p* = 0.357), outdoor track running (χ2_2_ = 0.245, *p* = 0.620), outdoor rural running (χ2_2_ = 0.158, *p* = 0.691), outdoor urban running (χ2_2_ = 1.302, *p* = 0.254), outdoor grass running (χ2_2_ = 0.202, *p* = 0.653), or changes in routes (χ2_2_ = 3.364, *p* = 0.067) (Table 5).

**Table 5:** Absolute changes in environment factors.

**Change**	**Group**	**Changes in environmental running behaviors (%)**							
		**Treadmill**	**Indoor track**	**Outdoor track**	**Rural road**	**Urban road**	**Trails**	**Grass**	**Routes**
Overall changes	Injured	33.3	5.1	21.2	41.4	57.6	**45.5**	9.1	45.5
	Uninjured	35.3	7.6	19.1	39.4	51.6	**32.8**	7.8	40.3
Increases	Injured	8.1	1.0	5.1	25.3	39.4	**25.3**	5.1	45.5
	Uninjured	7.1	0.4	4.6	27.2	36.0	**18.6**	5.1	19.5
Decreases	Injured	25.3	4.0	16.2	16.2	18.2	20.2	4.0	0.0
	Uninjured	28.2	7.2	14.5	12.2	15.5	14.2	2.7	20.9
No change	Injured	66.7	94.9	78.8	58.6	42.4	54.5	90.9	44.4
	Uninjured	64.7	92.4	80.9	60.6	48.4	67.2	92.2	59.1

*Injured refers to individuals who sustained a new RRI during COVID-19 restrictions. Uninjured refers to individuals who did not sustain a new RRI during COVID-19 restrictions*.

*Values in bold indicate significance (p < 0.05)*.

*Percentage change in environment-related running behaviors by injury group*.

When examining proportional changes in environment-related running behaviors, there was no significant difference between those with and without new injuries in proportional change in treadmill use (Z = −0.359, *p* = 0.719), indoor track running (Z = −1.312, *p* = 0.190), outdoor track running (Z = −0.176, *p* = 0.860), rural road running (Z = −1.090, *p* = 0.276), urban road running (Z = −0.971, *p* = 0.331), trail running (Z = −0.533, *p* = 0.594), and grass running (Z = −1.269, *p* = 0.205).

### Social-Related Running Behaviors

Regarding social running behaviors, we found no significant relationship between new injuries and changes in running alone strictly (χ2_2_ = 1.057, *p* = 0.304), running alone (χ^2^= 0.818, *p* = 0.366), running in a group (χ2_2_ = 0.781, *p* = 0.377), running with a dog (χ2_2_ = 2.135, *p* = 0.144), or running with a stroller (χ2_2_ = 1.911, *p* = 0.167).

### Barriers

We found a significant relationship between those that sustained a new injury and those that reported less time to exercise due to changes in work environment (χ2_1_ = 5.716, *p* = 0.017). No significant relationship was reported between injury and any additional barriers, including not having access to running group (χ2_1_ = 1.104, *p* = 0.294), apprehension in running alone (χ2_1_ = 0.457, *p* = 0.499), lacking motivation (χ2_1_ = 2.603, *p* = 0.107), stress or anxiety in leaving the home (χ2_1_ = 0.817, *p* = 0.366), less time obligations (χ2_1_ = 0.780, *p* = 0.378), limited access to running routes (χ2_1_ = 0.218, *p* = 0.641), limited access to safe environments (χ2_1_ = 3.574, *p* = 0.059), and having no access to workout facilities (χ2_1_ = 2.249, *p* = 0.134).

### Psychosocial Measures

**Figure 4:**
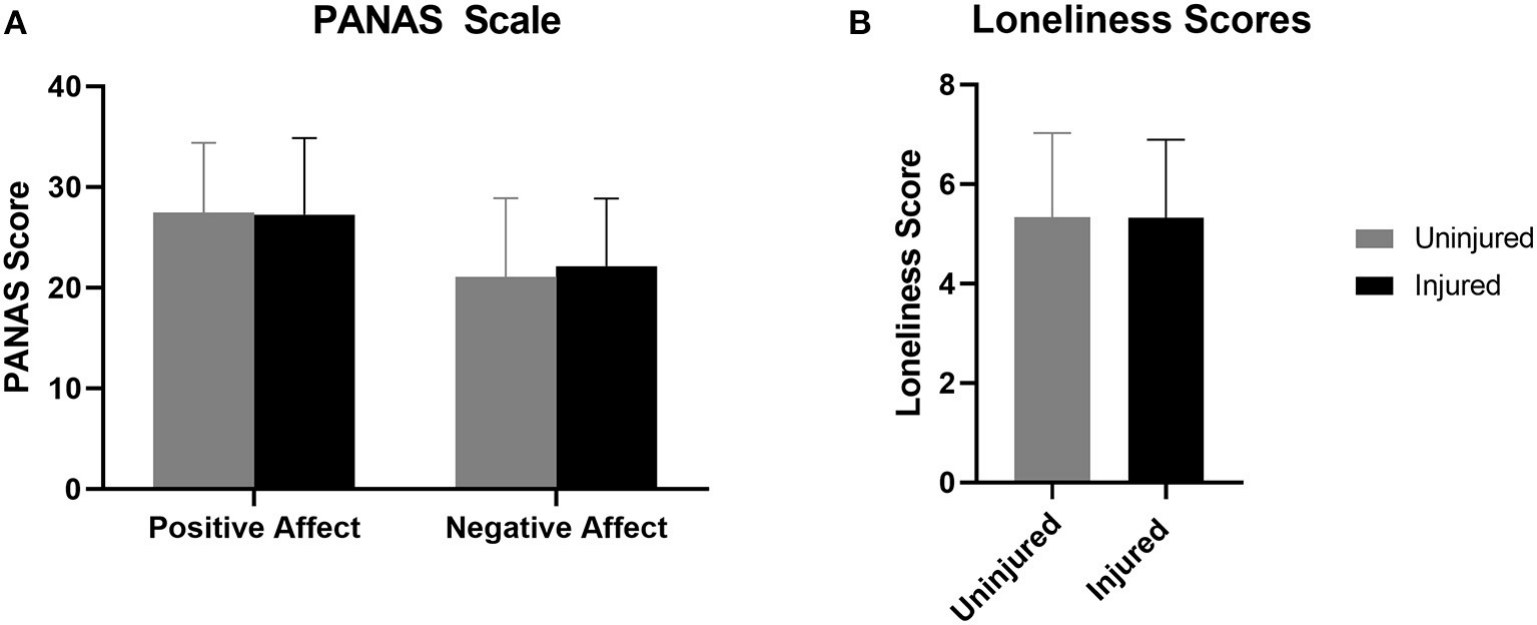
Injured refers to runners who sustained a new RRI during COVID-19 restrictions. Uninjured refers to runners who did not sustain a new RRI during COVID-19 restrictions. **(A)** Positive and negative affect and **(B)** measures of loneliness were not different between injured and uninjured runners.

We found no significant differences between those that sustained a new injury and uninjured runners in positive affect (Z = −0.494, *p* = 0.622), negative affect (Z = −1.622, *p* = 0.105), or loneliness scores (Z = −0.59, *p* = 0.953) (Figure 4). Loneliness scores were positively associated with total changes in running behaviors (*r* = 0.168, *p* <0.001, *R*^2^ = 0.028), as were negative affect scores (*r* = 0.072, *p* = 0.030, *R*^2^ = 0.005). Loneliness was positively correlated with training changes (*r* = 0.096, *p* = 0.002, *R*^2^ = 0.009), environmental changes (*r* = 0.141, *p* < 0.001, *R*^2^ = 0.020), and social changes (*r* = 0.119, *p* < 0.001, *R*^2^ = 0.014). There was no relationship between positive affect scores and total changes (*r* = −0.004, *p* = 0.913, R^2^ <.001). Positive affect scores were not related to training changes (*r* = −0.023, *p* = 0.476, *R*^2^ < 0.001), environmental changes (*r* = −0.015, *p* = 0.635, *R*^2^ < 0.001), or social changes (*r* = −0.052, *p* = 0.115, *R*^2^ = 0.002). Negative affect scores were not related to training changes (*r* = 0.039, *p* = 0.218, *R*^2^ = 0.002), environmental changes (*r* = 0.060, *p* = 0.063, *R*^2^ = 0.004), or social changes (*r* = 0.058, *p* = 0.078, *R*^2^ = 0.003).

The results of the OLS regression revealed that the combination of PANAS and loneliness scores significantly explained changes in light intensity running that were observed in response to COVID-19 restrictions [*F*_(3, 973)_ = 2.838, *p* = 0.037], and explained about 1% of the variance in proportional changes in light intensity running (*R*^2^ = 0.009, Adj. *R*^2^ = 0.006, SE = 0.257). Positive affect scores were a significant predictor (B = 0.081, β = −0.070, *t* = −2.115, *p* = 0.035) of proportional changes in light intensity running, indicating that higher positive affect scores were negatively related to proportional changes in light intensity running during COVID-19 restrictions. Neither negative affect scores (B = 0.000, β = 0.000, *t* = 0.004, *p* = 0.996) nor loneliness scores (B = 0.007, β = 0.048, *t* = 1.226, *p* = 0.221) were significant predictors. The combination of PANAS and loneliness scores did not significantly explain changes in hard intensity running [*F*_(3, 973)_ = 2.534, *p* = 0.056, *R*^2^ = 0.008, Adj. *R*^2^ = 0.005, SE = 0.218].

Loneliness scores were positively associated with apprehension to running alone (*r* = 0.163, *p* < 0.001, *R*^2^ = 0.027), lack of motivation (*r* = 0.237, *p* < 0.001, *R*^2^ = 0.056), and stress and anxiety of leaving home (*r* = 0.170, *p* < 0.001, *R*^2^ = 0.029). Positive affect scores were negatively associated with lack of motivation (*r* = −0.320, *p* < 0.001, *R*^2^ = 0.102), and stress and anxiety of leaving home (*r* = −0.140, *p* < 0.001, *R*^2^ = 0.020). Negative affect scores were positively associated with apprehension to running alone (*r* = 0.134, *p* < 0.001, *R*^2^ = 0.018), lack of motivation (*r* = 0.135, *p* < 0.001, *R*^2^ = 0.018), and stress and anxiety of leaving home (*r* = 0.313, *p* < 0.001, *R*^2^ = 0.10).

## Discussion

We report three main findings: (1) the total number of changes in running behaviors, regardless of the category of running behavior, and direction of change (increase or decrease), and changes in training and environmental running behaviors were associated with the occurrence of RRI, (2) less time to exercise, due to changes in work environment, was associated with RRIs, and (3) feelings of loneliness and positive and negative affect were associated with running behaviors and barriers linked to RRI.

### Changes in Running Behaviors

Considering all categories of running behaviors (training, environmental, and social), we observed that a greater number of total changes in running behavior were associated with the occurrence of RRIs. The total changes are reflective of a combination of increases or decreases across the various categories of running behaviors (training, environmental, or social running behaviors). Total changes averaged 8.2 for injured runners, whereas uninjured runners averaged 7.4 total changes. Analyzing changes in running frequency, distance, and duration in isolation did not reveal any significant associations with RRIs. Furthermore, although the total changes were significantly different between injured and uninjured runners, the difference was, on average, approximately one behavior change. Therefore, the lack of differences among commonly reported running behaviors (frequency, distance, and duration), along with the sensitivity in total running behavior changes across training, environmental, and social behaviors, support the need to analyze RRIs in a more complex framework with many interacting components. The interaction of commonly measured running behaviors (frequency, intensity, and duration) is crucial when investigating RRIs (Nielsen et al., 2012; Bittencourt et al., 2016; Bertelsen et al., 2017). Prior research has shown that changes in training-related running behaviors may inadvertently predispose individuals to injury, while external parameters such as running surface have also been shown to influence RRIs (Buist et al., 2010; Nielsen et al., 2013). Our study outcomes reinforce recent findings from Fredette and colleagues, who reported that 75.7% of Canadian military members that suffered an RRI had made recent changes to their running behavior, just prior to injury onset (Fredette et al., 2020). Therefore, our results build upon previous findings, highlighting the need to analyze RRI risk in a complex framework, incorporating a range of training, environment, and social domains of running behaviors and internal and external factors like psychological well-being and barriers to running routines.

We observed that training and environment changes, but not social changes, were associated with the occurrence of new RRIs sustained during COVID-19 restrictions. Running intensity was the only training-related running behavior that influences the occurrence of new RRIs. In fact, ~50% more of our participants that sustained new RRI, compared with uninjured participants, increased the number of maximal intensity per week. Although high-intensity running has been previously associated with RRIs, the reporting of intensity is inconsistent (Nielsen et al., 2012). Studies identifying how running intensity influences RRIs commonly use running pace as an indicator of intensity (Nielsen et al., 2012). In our study, participants ranked perceived intensity from light, moderate, hard, and maximal. Despite the participants that sustained a new RRI reporting an increase in the number of weekly maximal intensity workouts (Figure 2D), injured participants also decreased the proportion of hard workouts and increased the proportion of light intensity workouts significantly more than uninjured runners (Figure 3). Newly injured runners, on average, decreased the proportion of hard intensity workouts by 3.3%, whereas uninjured runners increased by a slight 0.1%. Furthermore, runners that experienced a new injury increased the proportion of light intensity workouts by ~9%, whereas uninjured runners only increased by ~4%. Before COVID restrictions, there were no significant differences in any training behaviors between injured and uninjured runners. Our findings on intensity may be a product of study design. For example, it is possible that newly injured runners depicted increases in the proportion of light intensity runs in response to injury. However, our results are consistent with previous literature relating RRI with increased intensity. Specifically, identifying the injured group's increase in the number of maximal intensity workouts per week corroborates prior literature depicting an increased risk of RRI with higher-intensity running (Jacobs and Berson, 1986; Hootman et al., 2002; Nielsen et al., 2012).

Regarding environmental changes in running behavior, more injured runners reported either increasing or not changing the number of runs per week they ran on trails. In fact, 25.3% of injured runners reported increasing the number of runs per week on trails compared with 18.6% of uninjured runners. Considering that surface type is vital in considering external load factors, different surfaces can alter mechanics (Dixon et al., 2000; Bertelsen et al., 2017). Differences among surface types are associated with alterations in ground reaction forces, leg stiffness, and joint kinematics during running (Dixon et al., 2000). Furthermore, running on a grass surface, as opposed to asphalt or concrete can reduce peak pressure in the rearfoot by 16% and by 12% in the forefoot (Tessutti et al., 2012). Previous work has shown that running on concrete and asphalt can influence injury risk, showing that runners with back and thigh injuries spent less percentage of their training running on concrete and asphalt surfaces (Macera et al., 1989; Wen et al., 1997). Specifically, well-trained female runners exhibit a decrease in average loading rate while running on rubber-modified asphalt (e.g., outdoor track surfaces) compared with running on conventional asphalt (Dixon et al., 2000). Trail running has been associated with RRIs, with one study reporting one in five trail runners injured at one time (Hespanhol Junior et al., 2017). Interestingly, our injured and uninjured runners were no different in the changes of the proportion of workouts run on each surface, indicating that the absolute change, rather than the change in the proportion of trail running days relative to all running days, may be of importance when considering RRIs.

### Barriers to Running

In addition to changes in running intensity and running surface types, less time to run due to changes in the work environment was significantly associated with the occurrence of RRIs. In fact, 56% of injured runners reported that they had less time to run due to changes in work environments. Our findings support recent work that examined adults' exercise levels in response to the COVID-19 lockdown, in which adults reported lack of time as a primary contributor to decreased activity levels (Constandt et al., 2020). Furthermore, the lack of time has also been a significant external barrier to exercise among University students (Gómez-López et al., 2010). Perhaps the lack of time to run due to work changes is associated with the increased total number of maximal-intensity runs observed in the injured runners. Due to time constraints, it is possible that runners sought to maximize the limited time they have by increasing the intensity of their runs. This could perhaps help explain why we observe an increase in the absolute number of maximal-intensity runs.

### Affect and Loneliness

Contrary to our hypothesis, running-related injuries were not associated with positive and negative affect or loneliness scores. However, we did find associations between these psychosocial measures and changes in running behavior. Our results indicate that negative affect and loneliness scores were associated with a greater number of total changes in running behavior, while there was no such association with positive affect scores. These findings support recent literature highlighting alterations in physical activity behaviors in response to the COVID-19 outbreak and their association with increased adverse psychological outcomes (Duncan et al., 2020; Stanton et al., 2020). For example, it has been shown that those reporting a negative change in physical activity were more likely to have higher depression, anxiety, and stress (Stanton et al., 2020). In our study, we identified moderate effect relationships among positive affect and decreased lack of motivation, and between negative affect and increased stress and anxiety leaving the home, both with implications on psychological well-being and potential influences on physical activity behavior. Not only is it pertinent to consider the impact of alterations in physical activity behavior on measures of psychological well-being but also the relationship is bi-directional. It is plausible that subjective well-being and psychological well-being measures can also lead to changes in physical activity behavior. Therefore, it is crucial to consider both elements when analyzing running behavior change.

Strategies aimed at mitigating the spread of the virus, such as social isolation and physical distancing, shelter in place, and quarantine, despite being necessary measures to help combat the spread of the virus, can have adverse psychological impacts (Brooks et al., 2020; Duncan et al., 2020; Stanton et al., 2020; Tian et al., 2020; Wang et al., 2020). In our study, we identified small, but significant relationships among increased loneliness and increased stress and anxiety of leaving the home. Research examining the immediate psychological response to the outbreak of COVID-19 reports that 70% of participants experienced moderate–severe psychological symptoms (Tian et al., 2020). Further literature depicts that 33% of the respondents report having moderate to severe anxiety in response to the COVID-19 outbreak (Wang et al., 2020). These psychological symptoms can directly impact negative affect, which describes experiencing the world in a negative manner and is highly influenced by stress and anxiety (McIntyre et al., 1990; Magyar-Moe, 2009). Therefore, the negative psychological impact of the COVID-19 global pandemic may indirectly influence RRIs by impacting the overall number of running behavior changes.

Loneliness scores were positively associated with changes in training, environmental, and social behaviors. Social network processes and social interactions with peers and friends are highly influential factors in determining physical activity behaviors (Macdonald-Wallis et al., 2012; Montgomery et al., 2020). However, strategies to combat COVID-19 have largely compromised these factors, through the closure of public workout facilities and limiting interactions with those outside of one's household. Therefore, such policies may have caused alterations in running behaviors and forced individuals to adapt to the unprecedented circumstances. It is possible that the resultant social isolation and subsequent alterations in training, environmental, and social running behaviors may have contributed to increasing the risk of RRI.

Therefore, when examining the underlying mechanisms of running-related injuries, it is crucial to consider measures of psychological well-being. In the face of stress or adversity, such as COVID-19, or other major life stressors, many turn to exercise as means of coping strategy (Cairney et al., 2014). The moderating role of emotional competencies has the ability to influence various aspects of running behavior.

### Limitations

This study comes with limitations. Through the nature of the survey design, it is possible that running injuries themselves influenced changes in running behaviors. We addressed this by analyzing training-specific running variables prior to injury between injured and uninjured runners and assessing questions that addressed COVID-19 as a specific influence on running behaviors. Running related injuries were self-reported. Although we provided a valid definition of RRI, a comprehensive list of common RRIs, and the ability to report an RRI not listed, accuracy of injury type has the potential to be compromised through self-report data. Because the dates of COVID-19 lockdowns varied by geographic location, we did not collect the date of injury. However, this limits our ability to interpret the duration that training changes may influence RRI risk. Due to the nature of the survey, we did not capture exact increases or decreases in training distance, frequency, and duration. Furthermore, training run intensity was based on self-report intensity without any physiological measurements (i.e., heart rate) or scales (i.e., Borg Scale Rate of Perceived Exertion). Due to the broad sample demographics and reflective nature of the survey (i.e., reflecting on running behaviors in the month preceding COVID-19 protocols), it is likely that not all participants track their exact training metrics or have tools to measure intensity, making this method of data sampling most appropriate. There are also other aspects of training (nutrition, hydration, sleep, footwear, recovery, and warm-ups) that were not included in this survey that could influence RRI risk and also are associated with changes induced by COVID-19. Future analyses should focus on how other potential influencers of RRI are affected by psychosocial and training behaviors. Because participants spanned many locations across the United States, there is a likely variability within surface types. For example, trail surfaces may vary on how perturbed the surface is, which may influence injuries. Furthermore, although COVID-19 restrictions may have been similar in different geographic locations, it is possible that some areas were of higher risk than others, which may make specific barriers inconsistent across participants. Due to the novelties that COVID-19 presents, this survey was broad with many statistical analyses to understand the scope of associations among running behaviors, psychological well-being, and running injuries. Our survey was novel with the intentions to test during this unique time. We did not have statistics on survey validity for the running behavior portion of the survey. Future analyses should validate survey parameters and focus on particular relationships to build upon the complex nature of RRI frameworks. Due to the exploratory nature of this study and group sample sizes, we did not adjust for multiple comparisons (Keppel and Wickens, 2007; Pituch and Stevens, 2016; Strunk and Mwavita, 2020). Thus, interpretations can be taken with caution. Follow-up studies and future investigations would benefit from such analyses on multiple comparisons. Furthermore, due to the novel complications of COVID-19 on health and lifestyle, there is a possibility that results of this study may not be fully generalizable to changes in running behavior and psychological well-being outside of COVID-19.

## Conclusion

A greater number of training, environmental, and social changes in running behaviors were associated with the occurrence of RRIs. The findings of our study highlight the importance of investigating all parameters of running (training-related, environment-related, social-related, and measures of psychological well-being), as opposed to isolating one specific aspect of running behavior. Our study highlights that individual barriers to exercise may also influence changes in running behavior. Understanding barriers associated with increased risk of injury may be useful for individuals undergoing various lifestyle changes. Runners may be forced to make multiple changes to their running routines dependent on life circumstances (i.e., moving, adjusted hours at work, new life stresses). Measures of psychological well-being were associated with running behavior and linked to RRI. Consideration of measures of psychological well-being are merited as they may serve a moderating role with respect to alterations in running behavior. Our findings highlight the importance of taking a multifactorial approach when examining RRI and suggests that exploring the impact of multiple changes may be more pertinent to garner a greater understanding of the causal mechanisms of RRI.

## Data Availability Statement

The raw data supporting the conclusions of this article will be made available by the authors, without undue reservation.

## Ethics Statement

Participants provided consent via agreeing to the informed consent document online. The study, informed consent and method of obtaining informed consent was approved by Auburn University's Institutional Review Board.

## Author Contributions

HH, PM, and JR conceptualized the research question. HH, PM, MP, and JR distributed the survey to the participants. HH and PM wrote the first draft of the manuscript. KS conducted the statistical analysis. KS, MP, and JR contributed to the manuscript with their expertise, and read, edited, and approved the final version of manuscript. All authors contributed to the article and approved the submitted version.

## Conflict of Interest

The authors declare that the research was conducted in the absence of any commercial or financial relationships that could be construed as a potential conflict of interest.
