# Supplementary material for: Changes in Training, Lifestyle, Psychological and Demographic Factors, and Associations With Running-Related Injuries During COVID-19
Source: Front Sports Act Living. 2021 Jun 7;3:637516. doi: 10.3389/fspor.2021.637516 (PMC8215167; doi:10.3389/fspor.2021.637516)
Supplement: Supplementary file 1 [file Data_Sheet_1.docx]

**Supplementary Data Sheet**

*Running behavior survey questions*

| **Training-Related Running Behaviors** | | | |
| --- | --- | --- | --- |
| Currently during COVID-19: | | Prior to COVID-19: | |
|  | How many times do you run each week?   - 0 times - 1-2 times - 3-4 times - 5-6 times - 7-8 times - > 8 times |  | How many times did you run each week?   - 0 times - 1-2 times - 3-4 times - 5-6 times - 7-8 times - > 8 times |
|  | How many minutes do you spend on your longest run?   - 0-10 minutes - 10-20 minutes - 20-30 minutes - 30-45 minutes - 45-60 minutes - 60-90 minutes - > 90 minutes |  | How many minutes did you spend on your longest run?   - 0-10 minutes - 10-20 minutes - 20-30 minutes - 30-45 minutes - 45-60 minutes - 60-90 minutes - > 90 minutes |
|  | How many miles do you average in a week?   - 0-10 miles - 10-20 miles - 20-40 miles - 40-60 miles - 60-80 miles - > 80 miles |  | How many miles did you average in a  week?   - 0-10 miles - 10-20 miles - 20-40 miles - 40-60 miles - 60-80 miles - > 80 miles |
|  | How many times per week do you run at a light intensity? |  | How many times per week did you run at a light intensity? |
|  | How many times per week do you run at a moderate intensity? |  | How many times per week did you run at a moderate intensity? |
|  | How many times per week do you run at a hard intensity? |  | How many times per week did you run at a hard intensity? |
|  | How many times per week do you run at a maximal intensity? |  | How many times per week did you run at a maximal intensity? |
| **Environment-Related Running Behaviors** | | | |
| Currently during COVID-19: | | Prior to COVID-19: | |
|  | Do you run different routes on most runs? (Yes/No) |  | Did you run different routes on most  runs? (Yes/No) |
|  | Do you run different routes on some runs? (Yes/No) |  | Did you run different routes on some  runs? (Yes/No) |
|  | Do you run the same routes when you run? (Yes/No) |  | Did you run the same routes when you  ran? (Yes/No) |
| How many times per week do you run in the following locations? | | How many times per week did you run in the following locations? | |
|  | Inside (treadmill) |  | Inside (treadmill) |
|  | Inside (track) |  | Inside (track) |
|  | Outside (track) |  | Outside (track) |
|  | Outside (roads, rural) |  | Outside (roads, rural) |
|  | Outside (roads, urban) |  | Outside (roads, urban) |
|  | Outside (trails)  Outside (grass) |  | Outside (trails) |
|  |  |  | Outside (grass) |
|  | **Social-Related Running Behaviors** | | |
|  | Currently during COVID-19: |  | Prior to COVID-19: |
|  | Do you run with members of your household? (Yes/No)  Do you run with friends/teammates/running group? (Yes/No)  Do you run inside on a treadmill? (Yes/No)  Do you alone? (Yes/No) |  | Did you run with members of your household? (Yes/No)  Did you run with friends/teammates/running group? (Yes/No)  Did you run inside on a treadmill? (Yes/No)  Did you alone? (Yes/No) |
| **Barriers to Running** | | | |
|  | No access to workout facilities (Yes/No)  Limited/no access to normal running routes (Yes/No)  Limited/no access to safe running environment (Yes/No)  Stress or anxiety related to leaving home (Yes/No)  Lack of motivation (Yes/No)  Apprehension to running alone (Yes/No)  Less time to exercise due to changes in work environment (Yes/No)  Less time to exercise due to added obligations at home (Yes/No) | | |
