# Supplementary material for: Changes in Training, Lifestyle, Psychological and Demographic Factors, and Associations With Running-Related Injuries During COVID-19
Source: Front Sports Act Living. 2021 Jun 7;3:637516. doi: 10.3389/fspor.2021.637516 (PMC8215167; doi:10.3389/fspor.2021.637516)
Supplement: Supplementary file 2 [file Data_Sheet_2.docx]

Survey Questions

How old are you?

▼ 18 ... 110 (93)

What was your sex assigned at birth?

- Male
- Female
- Intersex
- Prefer not to say

Choose one or more races that you consider yourself to be:

- White
- Black or African American
- American Indian or Alaska Native
- Asian
- Native Hawaiian or Pacific Islander
- Other race not listed here ________________________________________________

Are you Spanish, Hispanic, or Latino or none of these?

- Yes
- None of these

Which statement best describes your employment status currently during COVID-19?

- Working (paid employee, full-time)
- Working (paid employee, part-time)
- Working (self-employed)
- Not working (temporary layoff from a job)
- Not working (looking for work)
- Not working (disabled)
- Student
- Retired
- Prefer not to answer
- Other ________________________________________________

Has work shifted to remote working?

- Yes, completely remote
- Partially remote
- No, nothing has changed

The following questions will concern the time period "During COVID-19," of which can be defined as the time since work changes, isolation procedures, area closures/restrictions, home-schooling etc. occurred in your area.

Do you currently run for exercise?

- Yes
- No

Display This Question:

If The following questions will concern the time period "During COVID-19," of which can be defined a... = No

Have you run for exercise in the past before COVID-19?

- Yes
- No

Skip To: End of Survey If Have you run for exercise in the past before COVID-19? = No

Display This Question:

If Have you run for exercise in the past before COVID-19? = Yes

And The following questions will concern the time period "During COVID-19," of which can be defined a... = No

When did you stop running?

- Less than a year ago
- 1-2 years ago
- 2-5 years ago
- 5-10 years ago
- 10-20 years ago
- More than 21 years ago

Skip To: End of Survey If When did you stop running? != Less than a year ago

Display This Question:

If When did you stop running? = Less than a year ago

And The following questions will concern the time period "During COVID-19," of which can be defined a... = No

And Have you run for exercise in the past before COVID-19? = Yes

Did you stop running due to changes induced by COVID-19?

- Yes
- No

Display This Question:

If The following questions will concern the time period "During COVID-19," of which can be defined a... = Yes

At what level of competition do you currently **run**?

- Recreational/fitness only, no racing
- Recreational fitness, road racing
- High School Team (Track/Cross-Country)
- Collegiate (DIII)
- Collegiate (DII)
- Collegiate (DI)
- Post-Collegiate - regional level
- Post-Collegiate - national level
- Post-Collegiate - international level
- Post-Collegiate - professional
- I compete in a different sport (please indicate sport and level) ________________________________________________

What is the highest level of competition you have **run** in your lifetime?

- Recreational/fitness only, no racing
- Recreational/fitness road racing
- High School Team (Track/Cross-Country)
- Collegiate (DIII)
- Collegiate (DII)
- Collegiate (DI)
- Post-collegiate - regional level
- Post-collegiate - national level
- Post-collegiate - international level
- Post-collegiate - professional
- I competed in a different sport (please indicate sport and level) ________________________________________________

Did you start running due to COVID-19 lifestyle changes?

- Yes
- No

Currently during COVID-19, how many times do you run in a week?

- 1 time
- 2-3 times
- 4-5 times
- 6-7 times
- 7-8 times
- >8 times

Currently during COVID-19, around how many minutes do you spend on your longest runs?

- 0-10 minutes
- 10-20 minutes
- 20-30 minutes
- 30-45 minutes
- 45-60 minutes
- 60-90 minutes
- > 90 minutes

Currently during COVID-19, how many miles do you average in a week?

- 0-10 miles
- 10-20 miles
- 20-40 miles
- 40-60 miles
- 60-80 miles
- >80 miles

Currently during COVID-19, do you run on different routes or the same routes?

- I run different routes on most runs
- I run different routes on some runs
- I run the same routes when I go run

Currently during COVID-19, how many times per week do you run at the following intensities? 0 indicates 0 times and 8 indicates greater or equal to 8 times.

|  | 0 | 1 | 2 | 3 | 5 | 6 | 7 | 8 |
| --- | --- | --- | --- | --- | --- | --- | --- | --- |

| Light () | 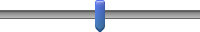 |
| --- | --- |
| Moderate () | 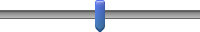 |
| Hard () | 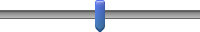 |
| Maximal () | 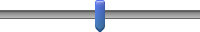 |

Currently during COVID-19, do you run with people, objects or animals? Please choose all that apply.

- I run with members of my household
- I run with friends/teammates/running group
- I run with with a stroller
- I run with my dog
- No, I run alone
- No, I run inside on a treadmill

Currently during COVID-19, how many times per week do you run in the following locations? 0 indicates 0 times and 8 indicates greater or equal to 8 times. Please choose all that apply.

|  | 0 | 1 | 2 | 3 | 4 | 5 | 6 | 7 | 8 |
| --- | --- | --- | --- | --- | --- | --- | --- | --- | --- |

| Inside (treadmill) () | 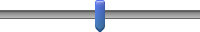 |
| --- | --- |
| Inside (indoor track) () | 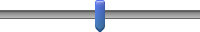 |
| Outside (track) () | 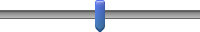 |
| Outside (road, rural) () | 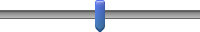 |
| Outside (road, urban) () | 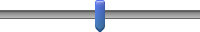 |
| Outside (trails) () | 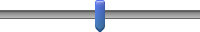 |
| Outside (grass) () | 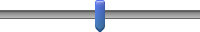 |

What new barriers to running has COVID-19 introduced?

- No access to workout facilities
- Limited/no access to normal running routes
- Limited/no access to open, safe environments
- Stress or anxiety related to leaving the home
- Lack of motivation
- Apprehension of running alone
- Less time due to changes in work environment (remote working, increased job hours etc)
- Less time due to added obligations at home (care-taker, etc)
- I experienced a running related injury
- I have not experienced any barriers
- Other _______________________________________________

Do you have a history of running injuries? NOTE: Here running-related injuries are defined as musculoskeletal pain in the lower limbs that causes a restriction on or stoppage of running (distance, speed, duration, or training) for at least 7 days or 3 consecutive scheduled training sessions, or that requires the runner to consult a physician or other health professional.

- Yes
- No

Display This Question:

If Do you have a history of running injuries? NOTE: Here running-related injuries are defined as mus... = Yes

What running injury do you have a history of?

- Lower back
- Pelvis - gluteus injury
- Pelvis-Sacral stress fracture/reaction
- Pelvis - other ________________________________________________
- Hip-Femoral neck stress fracture/reaction
- Hip - other ________________________________________________
- Thigh femoral stress fracture/reaction
- Thigh-other ________________________________________________
- Knee-Patellofemoral pain syndrome
- Knee-Meniscus injury
- Knee - Patellar tendinopathy
- Knee - Iliotibial band syndrome
- Knee - other ________________________________________________
- Lower leg - tibial stress fracture/reaction
- Lower leg - other ________________________________________________
- Ankle - Achilles tendinopathy
- Ankle - calf strains
- Ankle - other ________________________________________________
- Foot - other ________________________________________________
- Tibialis posterior tendinopathy
- Piriformis syndrome
- SI joint dysfunction
- Hamstring strain
- Morton's Neuroma
- Other ________________________________________________

During COVID-19, have you gotten injured running?

- Yes
- No

What running injury do you have during COVID-19?

- Lower back
- Pelvis - gluteus injury
- Pelvis-Sacral stress fracture/reaction
- Pelvis - other ________________________________________________
- Hip-Femoral neck stress fracture/reaction
- Hip - other ________________________________________________
- Thigh femoral stress fracture/reaction
- Thigh-other ________________________________________________
- Knee-Patellofemoral pain syndrome
- Knee-Meniscus injury
- Knee - Patellar tendinopathy
- Knee - Iliotibial band syndrome
- Knee - other ________________________________________________
- Lower leg - tibial stress fracture/reaction
- Lower leg - other ________________________________________________
- Ankle - Achilles tendinopathy
- Ankle - calf strains
- Ankle - other ________________________________________________
- Foot - other ________________________________________________
- Tibialis posterior tendinopathy
- Piriformis syndrome
- SI joint dysfunction
- Hamstring strain
- Morton's Neuroma
- Other ________________________________________________

During COVID-19, how often do you feel:

|  | Hardly Ever | Some of the Time | Often |
| --- | --- | --- | --- |
| Feel that you lack companionship |  |  |  |
| Feel left out |  |  |  |
| Feel isolated from others |  |  |  |

| Indicate the extent you have felt this way during COVID-19: | Very slightly or not at all | A little | Moderately | Quite a lot | Extremely |
| --- | --- | --- | --- | --- | --- |
| Interested |  |  |  |  |  |
| Distressed |  |  |  |  |  |
| Excited |  |  |  |  |  |
| Upset |  |  |  |  |  |
| Strong |  |  |  |  |  |
| Guilty |  |  |  |  |  |
| Scared |  |  |  |  |  |
| Hostile |  |  |  |  |  |
| Enthusiastic |  |  |  |  |  |
| Proud |  |  |  |  |  |
| Irritable |  |  |  |  |  |
| Alert |  |  |  |  |  |
| Ashamed |  |  |  |  |  |
| Inspired |  |  |  |  |  |
| Nervous |  |  |  |  |  |
| Determined |  |  |  |  |  |
| Attentive |  |  |  |  |  |
| Jittery |  |  |  |  |  |
| Active |  |  |  |  |  |
| Afraid |  |  |  |  |  |

The following questions refer to your running habits in the month leading up to COVID-19 (i.e. before remote working, before restrictions, before isolation protocols occurred in your area).

Before COVID-19, How many times a week did you run?

- 0 times
- 1 time
- 2-3 times
- 4-5 times
- 6-7 times
- > 8 times

Skip To: End of Survey If The following questions refer to your running habits in the month leading up to COVID-19 (i.e. be... = 0 times

Before COVID-19, around how many minutes did you spend on your longest runs?

- 0-10 minutes
- 10-20 minutes
- 20-30 minutes
- 30-45 minutes
- 45-60 minutes
- 60-90 minutes
- > 90 minutes

Before COVID-19, how many miles did you average in a week?

- 0-10 miles
- 10-20 miles
- 20-40 miles
- 40-60 miles
- 60-80 miles
- >80 miles

Before COVID-19, did you run outside with other people, objects or animals? Please choose all that apply

- I ran with members of my household
- I ran with friends/teammates/running group
- I ran with a stroller
- I ran with my dog
- No, I ran alone
- No, I ran inside on a treadmill

When you ran with others, on average how many people did you run with?

- 1-2 people
- 3-4 people
- > 5 people

Before COVID-19,  did you run on different routes or the same routes?

- I ran different routes on most runs
- I ran different routes on some runs
- I ran the same routes when I ran

Before COVID-19, were the location of your running routes the same as they are now?

- Yes, most of my runs are in the same locations
- Some of my runs are in the same locations
- No, I have had to switch locations of most all my runs

Prior to COVID-19, how many times per week did you run in the following locations? 0 indicates 0 times and 8 indicates 8 or more times. Please Choose all that apply.

|  | 0 | 1 | 2 | 3 | 4 | 5 | 6 | 7 | 8 |
| --- | --- | --- | --- | --- | --- | --- | --- | --- | --- |

| Inside (treadmill) () | 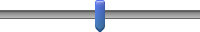 |
| --- | --- |
| Inside (indoor track) () | 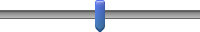 |
| Outside (track) () | 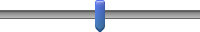 |
| Outside (road, rural) () | 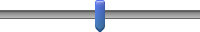 |
| Outside (road, urban) () | 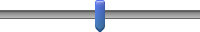 |
| Outside (trails) () | 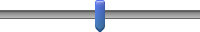 |
| Outside (grass) () | 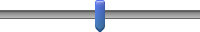 |

Prior to COVID-19, how many times per week would you run at the following intensities? 0 indicates 0 times and 8 indicates 8 or more times.

|  | 0 | 1 | 2 | 3 | 4 | 5 | 6 | 7 | 8 |
| --- | --- | --- | --- | --- | --- | --- | --- | --- | --- |

| Light () | 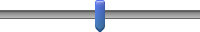 |
| --- | --- |
| Moderate () | 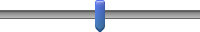 |
| Hard () | 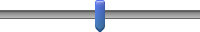 |
| Maximal () | 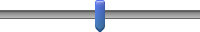 |
